# *Mycobacterium tuberculosis* Co-operonic PE32/PPE65 Proteins Alter Host Immune Responses by Hampering Th1 Response

**DOI:** 10.3389/fmicb.2016.00719

**Published:** 2016-05-17

**Authors:** Mohd Khubaib, Javaid A. Sheikh, Saurabh Pandey, Battu Srikanth, Manish Bhuwan, Nooruddin Khan, Seyed E. Hasnain, Nasreen Z. Ehtesham

**Affiliations:** ^1^Inflammation Biology and Cell Signaling Laboratory, National Institute of PathologyNew Delhi, India; ^2^Dr. Reddy’s Institute of Life Sciences, University of Hyderabad CampusHyderabad, India; ^3^Department of Biotechnology, School of Life Sciences, University of HyderabadHyderabad, India; ^4^Molecular Infection and Functional Biology Laboratory, Kusuma School of Biological Sciences, Indian Institute of TechnologyNew Delhi, India

**Keywords:** *M. tuberculosis*, PE32/PPE65, CD4^+^ T cells, CD8^+^ T cells, IgG subtyping

## Abstract

*PE/PPE* genes, present in cluster with *ESAT-6* like genes, are suspected to have a role in antigenic variation and virulence of *Mycobacterium tuberculosis.* Their roles in immune evasion and immune modulation of host are also well documented. We present evidence that *PE32/PPE65* present within the RD8 region are co-operonic, co-transcribed, and co-translated, and play role in modulating host immune responses. Experiments with macrophage cell lines revealed that this protein complex suppresses pro-inflammatory cytokines such as TNF-α and IL-6 whereas also inducing high expression of anti-inflammatory IL-10. Immunization of mice with these recombinant proteins dampens an effective Th1 response as evident from reduced frequency of IFN-γ and IL-2 producing CD4^+^ and CD8^+^ T cells. IgG sub-typing from serum of immunized mice revealed high levels of IgG1 when compared with IgG2a and IgG2b. Further IgG1/IgG2a ratio clearly demonstrated that the protein complex manipulates the host immune response favorable to the pathogen. Our results demonstrate that the co-transcribed and co-translated PE32 and PPE65 antigens are involved specifically in modulating anti-mycobacterial host immune response by hampering Th1 response.

## Introduction

Tuberculosis (TB), caused by the bacterium *Mycobacterium tuberculosis* (*M. tuberculosis*), despite being completely curable, claimed more than 1.5 million human lives in 2013 alone (Zumla et al., 2015) and continue to do so. With over a third of the world population infected with *M. tuberculosis*, and 9 million new cases each year, the major problems with regard to TB are: (i) lack of proper diagnosis; (ii) unavailability of efficacious vaccine; (iii) nexus with HIV, and (iv) emergence of MDR and XDR strains which is further aggravating the problem. The current protective TB vaccine- a live-attenuated *M. bovis* Bacillus-Calmette-Guerin (BCG) vaccine is protective against TB and leprosy in young people, but it is not efficient in protecting adults against pulmonary TB or reactivation of the latent TB infection (Mangtani et al., 2014). Proteins unique to these bacteria will be of interest in understanding the pathobiology of this bacterium. Identification of virulence determinants and components that are predicted to interact with host immune system are important to understand the pathogenicity in order to develop new therapeutic interventions.

While mycobacteria underwent reductive evolution as it attained pathogenicity (Ahmed et al., 2008), the only protein family whose presence increased as the genome size decreased was the PE/PPE/PGRS family (Gey van Pittius et al., 2006), present exclusively in this genus (Cole, 1998). Apart from interspecies difference, clinical isolates of even single species depicted genome fluidity as a function of geographic partitioning (Rao et al., 2005). Comparative genomic and proteomic analyses of pathogenic, opportunistic and non-pathogenic mycobacteria revealed an inverse relationship between the number of genes and the pathogenicity/virulence status of the *Mycobacterium* (Rahman et al., 2014). While *M. tuberculosis* genome encodes about 175 PE/PPE proteins (Cole, 1998), the recently sequenced ancestral, non-pathogenic and medically relevant *M. indicus pranii* carries only 66 *PE/PPE* genes (Saini et al., 2009, 2012). These genes were speculated to be introduced into mycobacteria by mobile elements *via* lateral gene transfer and later evolved from esx-encoded ancestral *PE/PPE* copies by gene duplication of this cluster (Gey van Pittius et al., 2006).

Despite representing about 10% of the total genome of *M. tuberculosis*, the role of these highly expanded and enigmatic protein families is not fully understood (Akhter et al., 2012). Many members of these gene families have been described to have multiple functions (moonlighting) by gene cooption (Singh et al., 2014) as a consequence of genomic reduction. Several *PE/PPE* genes are co-operonic and are present in clusters along with ESX and/or other proteins (Tundup et al., 2006). ESX, also known as Type VII secretion system, is present in five copies and one of the ESX coding gene cluster has been shown to be essential for virulence (Houben et al., 2014). ESAT-6 and CFP-10, secreted out through the ESX secretory complex interacts with host factors and modulates the immune response (Delogu et al., 2006). This close evolutionary link between ESX clusters and *PE/PPE* genes along with key difference within this family of proteins, between the virulent (H_37_Rv) and avirulent (H_37_Ra) strain, suggests that PE/PPE proteins contribute to infection and pathogenesis (Kohli et al., 2012). These proteins have other myriad functions like, signal transduction (Strong et al., 2006; Deng et al., 2014) role in virulence (Choudhary et al., 2003), immune modulation (Parra et al., 2006), inhibition of phagolysosomal fusion (Jha et al., 2010) immune quorum sensing with subsequent cell death (Tundup et al., 2014), and antigenic variation (Banu et al., 2002; Mohareer et al., 2011). However, antigenic variations found in PE-PGRS family is now attributed to the selection pressure acting on individual *PE-PGRS* gene and independent of human T-cell recognition (Copin et al., 2014).

Comparative genomics of different species of *M. tuberculosis* complex revealed differences in few regions of genome, known as Region of Difference (RD). Role of most of the 16 RDs is not clearly understood except for RD1, which has been shown to be the most important factor for virulence (Lewis et al., 2003). RD1 has *PE35/PPE68* genes clustered along with ESAT-6 and CFP-10 and ESX secretory system which are known virulent factors of *M. tuberculosis* (Jackson-Sillah et al., 2013). Another RD harboring *PE/PPE* genes clustered with ESAT-6 system is RD8, which codes PE32/PPE65, tempting us to speculate that the *PE32/PPE65* antigens might be potential virulence determinants. Our results show that *PE32/PPE65* are co-operonic and are expressed together at mRNA as well as at protein levels. We used *in silico* approach to predict the antigenicity of PE32/PPE65 using hydropathy profiling which revealed strong antigenic index for PPE65 as compared to PE32. These *in silico* findings were experimentally validated *in vitro* and *in vivo* in a mouse model. Cytokine and IgG profiling data categorically reveal that these proteins have a role in immune modulation and aid in pathogenicity.

## Materials and Methods

### Cloning, Expression, and Purification of Recombinant Proteins

*PE32* and *PPE65* genes were amplified from H_37_Rv genomic DNA using specific primers (**Table 1**). The complete ORF encoding *PE32* and *PPE65* was cloned into *Bam*HI and *Xho*I site or *Bam*HI and *Hin*dIII site of pETDuet-1 vector (Novagen) to construct recombinant expression plasmids pET-PE32 and pET-PPE65, respectively. DNA fragment containing both the genes was PCR amplified from H_37_Rv genomic DNA using forward primer specific for PE32 and reverse primer specific for PPE65 and the resultant amplicon was cloned into the *Bam*HI and *Hin*dIII site of pETDuet vector to generate pET-PE32:PPE65. Recombinant constructs were used to transform *Escherichia coli* BL21-DE3 strain. Culture was induced with 1 mM Isopropyl β-D-1-thiogalactopyranoside (IPTG) for 3 h at 37°C for the expression of recombinant proteins. Purification of recombinant proteins was carried out by sonicating bacterial cells and solubilizing recombinant protein using 0.3% Sarkosyl in PBS. Recombinant proteins were purified using Ni-NTA affinity column and eluted with 200 mM Imidazole, dialyzed against PBS and finally treated with Polymyxin B beads to remove bacterial endotoxins.

**Table 1:** Different primers used in this study.

Primer	Sequence	Restriction enzyme site
PE32F (P1)	TCCAGATCTAATGTCGATCATGCACGCCGAGC	*Bgl*II
PE32R (P2)	GAACTCGAGCTAAGCGATCGTGGCGGCGT	*Xho*I
PPE65F (P3)	CCGGATCCTATGCTGGACTTTGCTCAGTTACCGC	*Bam*HI
PPE65R (P4)	CCGAAGCTTCGATCCTCGATCAACGAACGATGTTG	*Hin*dIII


### Operonic Organization

DNaseI treated total RNA of H_37_Rv was used to synthesize first strand cDNA, using specific reverse primer for *PPE65*. This single strand cDNA was used as template to amplify PE/PPE genes using gene specific primer pairs: (a) *PE32* forward and reverse; (b) *PPE65* forward and reverse; (c) *PE32* forward and *PPE65* reverse primer. **Table 1** lists the sequence of all the primers used in this study. Genomic DNA was placed as positive control and reaction without reverse transcriptase served as negative control.

To check for co-operonic expression of protein, DNA fragment containing both genes was amplified from H_37_Rv genomic DNA using forward primer specific for *PE32* and reverse primer specific for *PPE65*. Amplified DNA fragment was cloned in MCS-I of pETDuet-1 vector between *Bam*HI and *Hin*dIII sites. *BL21-DE3* expression strain of *E. coli* was transformed with the clone and proteins expressed as mentioned earlier.

### Immunization of Mice

Eight to twelve week old C57BL/6j mice were procured from National Institute of Nutrition, Hyderabad, India. All procedures were performed in accordance with Institutional Animal Ethics guidelines. Mice were housed in cages and allowed free access to pellet diet and water with 12 h photoperiod. Period of 7 days was given for acclimatization to the environment and observation for signs of disease. Primary immunization was carried out with 20 μg of recombinant PE/PPE or PE+PPE proteins in PBS, administered subcutaneously at the base of tail. Mice were checked daily for signs of discomfort. Booster was given subcutaneously with 20 μg of recombinant PE/PPE protein at 15th day of immunization. Mice were sacrificed after 4 weeks of primary immunization.

### Cell Culture and Estimation of Cytokines by ELISA

RAW264.7 cells were seeded (0.1 million/well) in 96-well plate. Cells were stimulated with different concentration of native or heat inactivated recombinant protein. After 24 h of stimulation, culture supernatant was harvested, and ELISA was performed as described earlier (Ravindran et al., 2014). Splenocytes from immunized and placebo treated mice were also seeded in 96-well plates (0.1 million/well) in DMEM. Protein treatments with different concentration were given for 72 h and supernatants were collected and stored at -20°C.

Secreted cytokine levels in culture supernatant were measured using OptEIA kits (BD Biosciences, San Diego, CA, USA) to determine the levels of mouse TNF-α, IFN-γ, IL-10, and IL-6. ELISA was carried out using manufacturer protocol or as described earlier (Ravindran et al., 2014). Briefly, 96 well ELISA plates were coated with capture antibody in coating buffer (bicarbonate/phosphate buffer) kept at 4°C overnight. Plates were washed with PBS-T (0.05% tween20) thrice. 10% FBS was used as blocking as well as assay diluent. After an hour of blocking, supernatant along with standards were added for 2 h. After 5 washes, detection antibody and enzyme conjugates were added for 1hr. After 7 washes, TMB substrate was added and 2N H_2_SO_4_ was added to stop the reaction. Absorbance was taken at 450 nm and curve was plotted along with standards to determine the cytokine levels in test samples.

B-cell response against individual proteins was carried out using specific secondary antibody against IgG1, IgG2a, and IgG2b. Blood was collected by bleeding mice *via* the lateral tail vein. Sera were collected and stored at -20°C to be used later for ELISA as described earlier (Banerjee et al., 2004). Briefly, 96 well plates were coated by specific proteins in PBS (10 μg/ml) and kept at 4°C overnight. Plate was washed three times with wash buffer and blocked for an hour at room temperature. After 3 washes serum samples in 1:100 dilution was added and kept for 2 h. Secondary conjugate antibody was added in 1:5000, 1:3000, and 1:3000, respectively, for IgG1, 2a and 2b for an hour (Kasturi et al., 2011). Plate was washed at least five times, TMB substrate was added, and reaction was stopped with 2N H_2_SO_4_.

### MTT Assay

MTT assay was carried out using RAW264.7 cells (1 × 10^4^/well) seeded in 96 well plates in complete DMEM media and treated with proteins in different concentrations for 24 h. Supernatant was harvested and fresh 200 μl media was added. 20 μl of MTT at a concentration of 5 mg/ml was added and incubated at 37°C for 4 h. 100 μl of DMSO was added to each well and mixed well after removing the media completely (Tundup et al., 2014). Absorbance was measured at 595 nm.

### Splenocyte Isolation and Single Cell Preparation

Mice were euthanized and spleens were isolated *via* incision at the left side of the abdomen. Spleens were crushed/perfused using syringe and the suspension was transferred to another tube after passing through cell strainer. Cells were centrifuged, washed and re-suspended in RBC lysis buffer. The cells were centrifuged and finally re-suspended in DMEM containing 10% FBS.

### Intracellular Cytokine (ICC) Staining and Extracellular Staining of Surface Markers

For CD4^+^/CD8^+^ T cell responses, 1 × 10^6^ Splenocytes/well were seeded in 96-well plate (Ravindran et al., 2014), and re-stimulated with 10 μg/ml concentration of PE32, PPE65 and PE32+PPE65 for 8 h in presence of Golgi plug^TM^ and Golgi stop^TM^ (BD Biosciences, San Diego, CA, USA). Cells were collected, washed with PBS and stained with anti-CD4 and anti-CD8 (FITC for CD4 and CD8). Cells were fixed with 4% paraformaldehyde, permeablized in 0.02% triton X-100, followed by washing and were stained with anti-IFN-γ and anti-IL-2 (APC for IFN-γ, PE for IL-2) for 1 h.

### Statistical Analysis

Statistical analysis was performed using Prism 5 software. Results were expressed as mean ± SD and analyzed by one-way ANOVA for calculating *p* value. Value of *p* < 0.05 was considered statistically significant.

## Results

### PE32 and PPE65 Are Organized in an Operon and Transcribed as a Single mRNA

**FIGURE 1:**
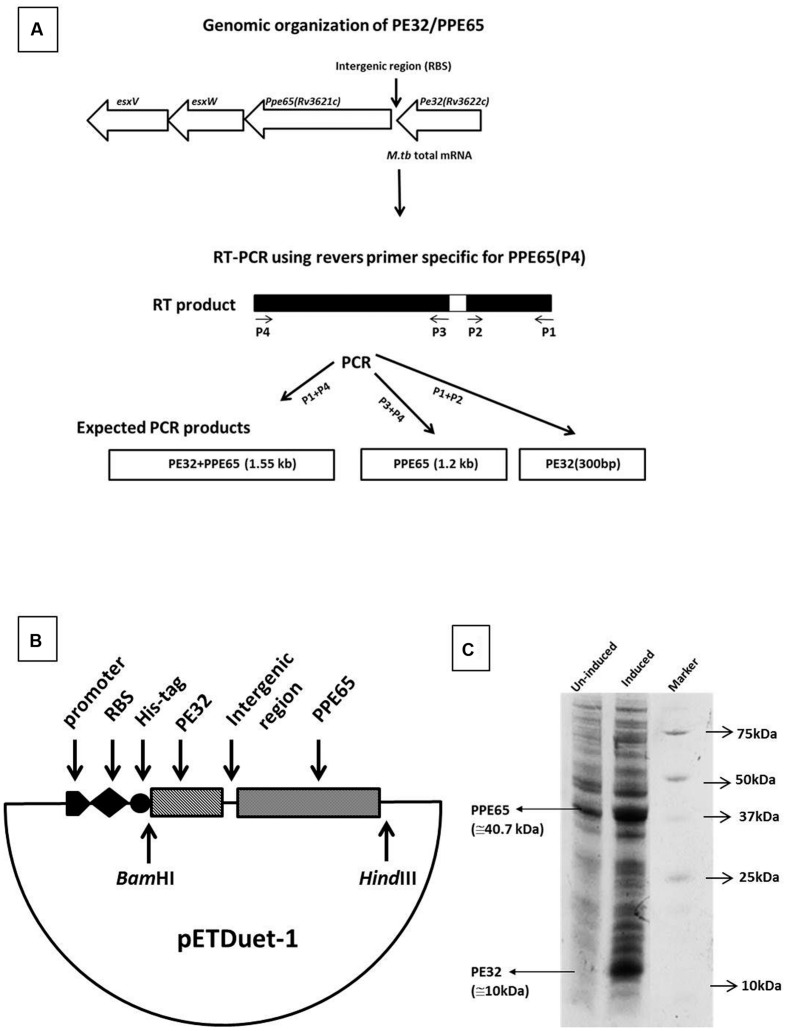
**Genomic organization and expression analysis reveals PE32 and PPE65 as functionally linked co-operonic gene pair.** *PE32/PPE65* (*Rv3622c/Rv3621c*) gene pair is organized in an operon. **(A)** Schematic representation of co-operonic genes. Primer pair specific to *PE32* produced amplicon of the size 300 bp, specific primer pair for *PPE65* yielded amplification of 1.2 kb, and forward primer for *PE32* and reverse primer for *PPE65* revealed an amplification product of 1.5 kb. **(B)** Schematic representation of clone of *PE32* + *PPE65* gene pair as single operon. **(C)** *PE32/PPE65* genes are expressed together at protein level. Expression of *PE32/PPE65* gene was checked on 10% tricine SDS gel. Lane 1 is un-induced culture, Lane 2 is induced culture showing the expression of PE32 (10 kDa) and PPE65 (40.7 kDa), Lane 3 is protein ladder.

*In silico* analysis predicted several *PE/PPE* genes, including the *PE32/PPE65* pair to be organized as operons in the genome (Tundup et al., 2006). RT-PCR was carried out using specific reverse and forward primers of *PPE65* and *PE32* for amplification of *PE32* (P1, P2), *PPE65* (P3, P4), and *PE32*+*PPE65* (P1, P4) were used (**Table 1**; **Figure 1A**). Agarose gel electrophoresis of the amplified product clearly shows amplification fragment of 300 bp corresponding to PE32, 1.2 kb of *PPE65*, and 1.5 kb of *PE32*+*PPE65* (**Supplementary Figure S1**). PCR reaction without reverse transcriptase with total RNA did not generate any amplification from genomic DNA contamination (**Supplementary Figure S1**). These results validated the *in silico* observation on the co-operonic expression of *PE32/PPE65* gene pair.

### Co-translation of PE32 and PPE65

Co-operonic *PE/PPE* genes have been earlier shown to be translated together when cloned in a vector having RBS (ribosome binding site) introduced in between (Strong et al., 2006; Tundup et al., 2006). Strong et al employed a co-expression vector that has been modified to include an additional RBS for expression of the second gene. However, we have not disturbed the intergenic region between the two genes, thus utilizing the RBS from vector for the expression of first gene (*PE32*). The native *M. tuberculosis* RBS present within the intergenic region was utilized for the second gene (*PPE65*). A cassette of *PE32*+intergenic region+*PPE65* was cloned under one T7 promoter with RBS and His tag fused to the *PE32* gene (**Figure 1B**). Assuming that a single mRNA will be transcribed for both the genes, RBS present in vector will carry out the translation of *PE32* while native RBS present before the *PPE65* gene will translate *PPE65*. This indeed could be seen when the protein expression product present in the supernatant of culture lysate was loaded on 10% tricine SDS gel. Over expression of PE32 (10 kDa) and PPE65 (40.7 kDa) can be observed (**Figure 1C**) as compared to un-induced culture. These data demonstrate that mRNA transcribed from the co-operonic PE32/PPE65 gene pair was translated using external RBS and the intergenic RBS to give rise to corresponding proteins PE32 and PPE65.

### PE32/PPE65 Proteins Reduce Pro-Inflammatory Response and Concomitantly Enhance Anti-Inflammatory Response

**FIGURE 2:**
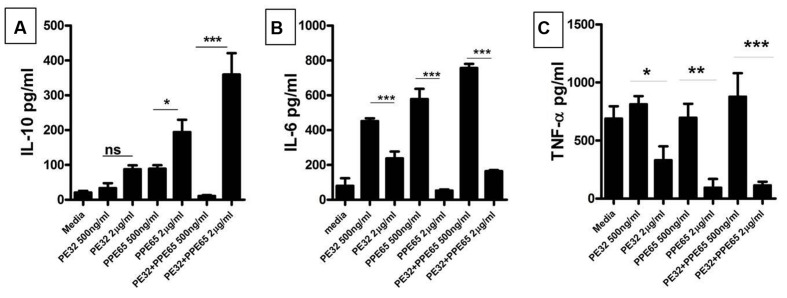
**PE32 and PPE65 modulate the host immune pathway by suppressing pro inflammatory cytokine production.** RAW 264.7 cells were treated with purified recombinant PE/PPE proteins in different concentration for 24 h, culture supernatants were collected for measuring various cytokine levels through sandwich ELISA. **(A)** PE32, PPE65 induce IL-10 level in RAW 264.7 cells as a direct function of protein concentration whether used singly or together. **(B,C)** Note the decrease in the level of Th1 cytokine (IL-6 and TNF-α) in RAW 264.7 cells with increase in protein concentration Experiments were performed at least twice; SEM is represented by error bar for biological triplicates. ^∗^*p* < 0.05, ^∗∗^*p* < 0.01, and ^∗∗∗^*p* < 0.001.

Recombinant PE32 and PPE65 were purified with N-terminal Histidine tag, with high purity (**Supplementary Figure S2**) and treated with Polymyxin B beads to remove endotoxins. Varying concentration of recombinant PE32, PPE65, and PE32+PPE65 proteins were used to stimulate RAW264.7 cells in culture for 24 h. PPE65 was shown to increase the level of anti-inflammatory cytokine IL-10 in a dose dependent manner in RAW264.7 cells (**Figure 2A**). PE32 alone also increased IL-10 production, though not significantly. Combinations of the two proteins also lead to significant elevation in IL-10 secretion. After observing increase in anti-inflammatory levels we evaluated pro-inflammatory response. IL-6 level was found to decrease (**Figure 2B**) as a direct function of increasing concentration of recombinant proteins either used singly or together in RAW264.7 cells. TNF-α secretion by RAW264.7 cells was found to decrease with increase in concentration of recombinant proteins (**Figure 2C**) To rule out cytotoxicity to be the reason for decrease in cytokine level with protein dose, we performed MTT assay which showed no significant death with higher protein dose (**Supplementary Figure S3**). These results demonstrate that PE32 and PPE65 proteins are immunomodulatory in nature and play role in either establishment or maintenance of infection to favor survival of pathogen by altering the cytokine milieu.

### B-Cell Response against PPE32/PPE65 in Immunized Mice

**FIGURE 3:**
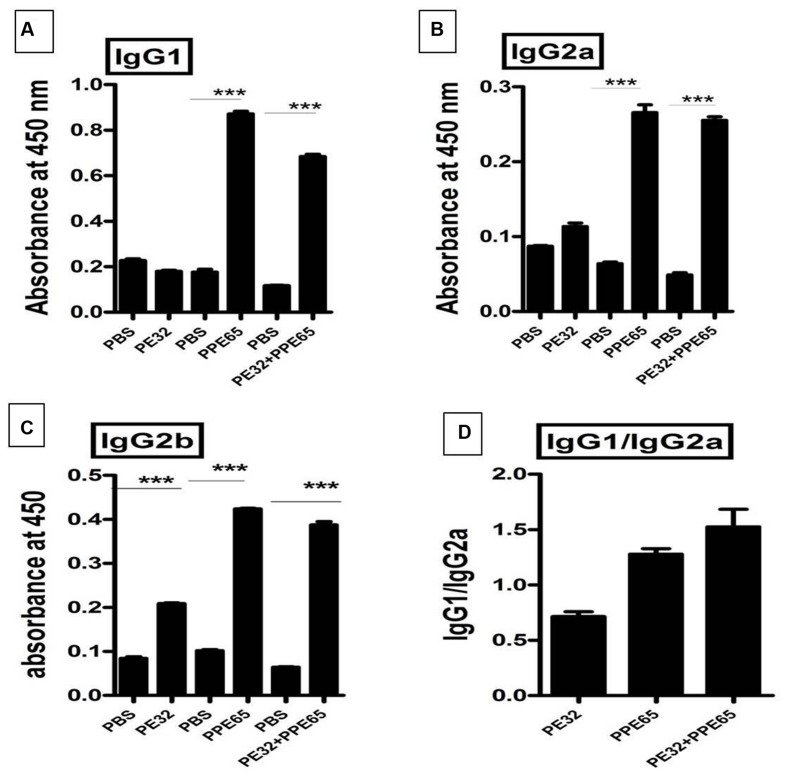
**Antibody isotype analysis reveals the skewing of immune phenotype toward Th2 type of response.** Sera from immunized mice were used to perform ELISA to measure IgG1, IgG2a, and IgG2b. **(A)** Mice immunized with PE32 show low IgG1 while those immunized with PPE65 and PE32 + PPE65 proteins showed higher levels of IgG1 against respective proteins. **(B,C)** Similar observation was made for IgG2a and IgG2b. IgG1 vs. IgG2a ratio is important to determine whether the response is Th1 or Th2. **(D)** The IgG1/IgG2a >1 shows Th2 response for PPE65 and PE32 + PPE65. Error bar represents SEM of average of three animals in biological triplicates. ^∗∗∗^*p* < 0.001.

Among several operonic PE/PPE proteins, PE had been seen to be less or non-immunogenic (Tundup et al., 2008) in mice as well as in humans. We used DNASTAR prediction tool (Choudhary et al., 2003) and expectedly found that PPE65 has numerous antigenic patches as compared to PE32 which had a few antigenic stretches indicating that PE32 could be less immunogenic (**Supplementary Figure S4**). *In vivo* antigenicity assay was carried out by immunizing mice with PE32, PPE65, and PE32+PPE65 followed by booster at 15^th^ day of primary immunization. Serum was collected after 4 weeks of primary immunization. ELISA was performed for IgG1, IgG2a, and IgG2b response against PE32, PPE65, and PE32+PPE65. IgG1 was the predominant subtype in immunized animal (3A) as compared to other subtypes (**Figures 3A–C**). Higher IgG2a and IgG2b response could also be observed in PPE65 and PE32+PPE65 immunized mice as compared to that of PE32 immunized mice which displayed low levels of all the isotypes (**Figures 3B,C**). Further, IgG1 and IgG2a ratio revealed that PPE65 and PE32+PPE65 induce Th2 response with no significant result seen for PE32 (**Figure 3D**). IgG isotype ratio clearly suggested predominance of Th2 response over Th1 that impairs anti-mycobacterial immunity.

### PE32/PPE65 Proteins Suppress Th1 Cytokines Production in Splenocytes

**FIGURE 4:**
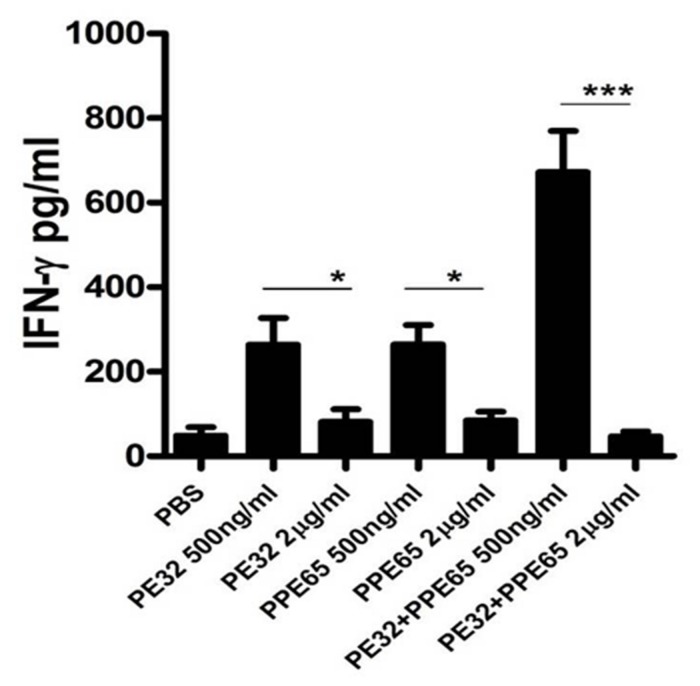
**PE32 and PPE65 proteins reduce the secretion of IFN-γ in splenocytes of mice immunized with respective proteins.** Splenocytes isolated from mice which were immunized with PE32, PPE65, and PE32 + PPE65 proteins were again challenged with corresponding proteins in culture for 72 h, supernatants were harvested and sandwich ELISA for IFNγ was performed. Note the decrease in IFNγ level with increase in protein concentration. Experiments were performed at least twice; SEM is represented by error bar for biological triplicates. ^∗^*p* < 0.05, ^∗∗∗^*p* < 0.001.

**FIGURE 5:**
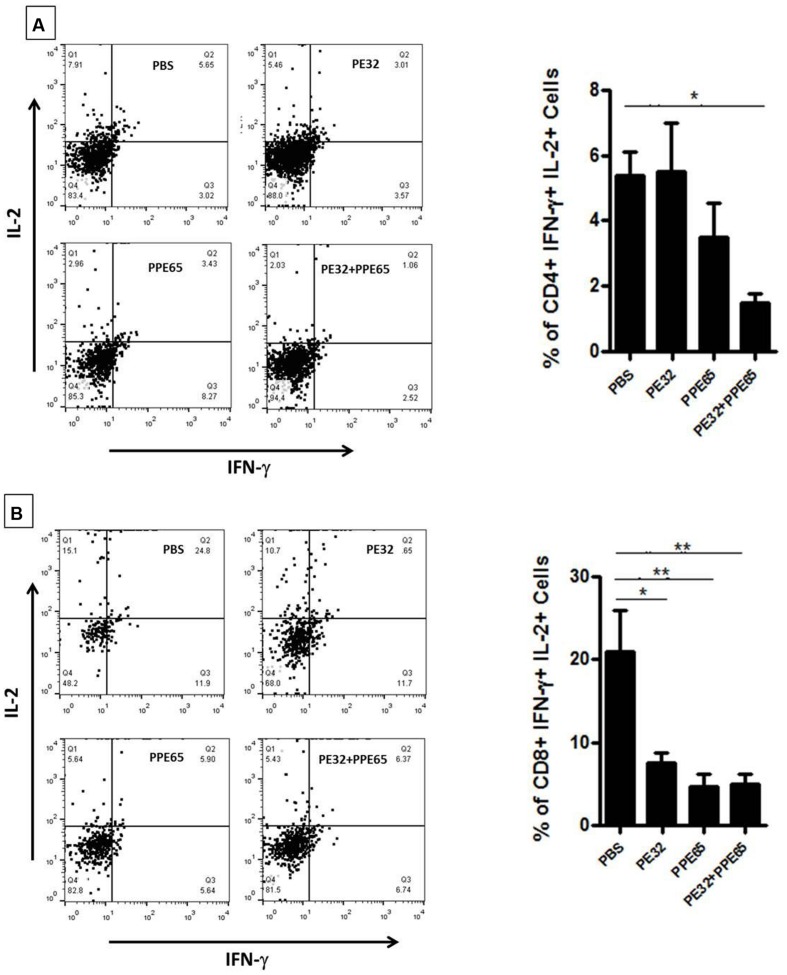
**PE32 and PPE65 proteins reduce polyfunctional CD4^+^ and CD8^+^ cells in immunized mice.** Splenocytes isolated from mice which were immunized with PE32, PPE65, and PE32 + PPE65 proteins were again challenged with same proteins in culture for 8 h and stained for intracellular IFN-γ and IL-2. **(A)** PE32 and PPE65 proteins decrease intracellular IFN-γ and IL-2 level as depicted by graphical representation of change in number of CD4^+^ cells positive for IFNγ and IL-2. **(B)** These proteins also decreased frequency of CD8^+^ cells expressing IFNγ and IL-2 as evident from graphical representation of change in frequency of CD8^+^ cells positive for IFNγ and IL-2. Dot plots are representative plots from at least four animals for each group. ^∗^*p* < 0.05, ^∗∗^*p* < 0.01.

Treatment of macrophage cell lines with PE32/PPE65 proteins demonstrated decrease in pro-inflammatory cytokines along with increase in anti-inflammatory cytokine. Result of IgG subtype responses also corroborated a shift toward Th2 type of immune response. Interestingly, animals immunized with these proteins either singly or in combination also depicted a decrease in IFN-γ level in splenocytes culture supernatant with increasing concentration of PE32, PPE65, and PE32+PPE65 (**Figure 4**). These finding directly point to the role of these proteins in dampening the anti-mycobacterial Th1 response. Having observed decrease in IFN-γ levels in culture supernatant of splenocytes treated with recombinant proteins, we assayed for T-cell activity by measuring CD4^+^/CD8^+^ cells positive for IFN-γ and IL-2 by flow cytometry. A decrease in CD4^+^ and CD8^+^ cells positive for IFN-γ and IL-2 could be seen after treatment with these PE/PPE proteins, with maximum decrease observed in splenocytes primed and treated with PE32+PPE65 (**Figures 5A,B**).

The immunological observations described above clearly indicate that this *PE/PPE* gene pair interacts with host components and thereby modulates critical immune pathways to subvert the immune response for the benefit of pathogen. This alteration of immune phenotype could profoundly impact the course of disease thus implicating this protein family as critical mediators of the host response that finally drives the outcome of infection.

## Discussion

The evolution of *M. tuberculosis* involves genomic reduction (Ahmed et al., 2008) with the exception of *PE/PPE/PGRS* family of genes which in fact have expanded as a function of evolution toward pathogenicity (Gey van Pittius et al., 2006). The organization of *PE/PPE* along with ESX family corroborates their role in evolution of pathogenicity and host specificity (Delogu et al., 2006; Tundup et al., 2006). Their unique organization with type VII secretion system makes them important targets of host immune response (Sampson, 2011). Although these proteins are reported to have direct role in pathogenesis and virulence (Ahmed et al., 2015: Fishbein et al., 2015) the actual mechanism is still obscure. We investigated the immunomodulatory behavior of *PE32/PPE65* gene complex organized within RD8 segment of *M. tuberculosis* genome. The operonic organization of these proteins suggests they are functionally linked and thus might have a common function with each protein enhancing the functionality of other one (Tundup et al., 2014). This observation supplements the information available regarding operonic organization of this important pathogen. The operonic organization suggests a functional linkage and thus probably a common biochemical pathways components that could be delineated and targeted to tame this pathogen. It can be speculated that these protein can function independently of each other as well as form a complex to have a gain of function. Our observation of increased effect by using the combination of both suggests that these protein possibly interact *in vivo* and adjunct the immunomodulatory function of each other.

Macrophages are the first line of defense against *M. tuberculosis* infection (Clay et al., 2007) but *M. tuberculosis* employs a plethora of strategies to counteract the host immunity, thereby successfully establishing a niche within macrophages. When RAW264.7 macrophage cell lines were treated with recombinant PE32/PPE65, either singly or in combination, they modulated immune response by decreasing production of pro-inflammatory cytokines and increasing anti-inflammatory cytokine production. Another protein of this family, PPE18 has earlier been reported to increase IL-10 production in THP-1 cells (Nair et al., 2009). Available data also indicate that PE/PPE proteins play important role in subverting innate immune responses and thus help the pathogen in establishment of infection. Apart from immune modulation many PE/PPE proteins have also been implicated in interference of vacuole acidification (Tiwari et al., 2012) which is a vital step for killing of these bacteria. Once bacteria establish itself in host macrophages by subverting innate immune defense it can then modulate the adaptive host immune mechanism for its survival (Goldstone et al., 2009). Apart from modulating the cytokine milieu PE/PPE proteins (PE_PGRS33 and PE_PGRS17) are known to assist in immune evasion by limiting antigen presentation (Delogu et al., 2004), thereby preventing recognition and killing of mycobacteria-infected host cells.

It is now established that B cells can exert influence on T cells and are thus likely to be important in determining the outcome of infection with *M. tuberculosis*. The IgG2a and the IgG1 isotypes level usually reflect the T cell phenotype. A Th2 biased response favors the production of IgG1 isotype and a Th1 response drives IgG2a production. As PE/PPE family members are a potentially rich source of B cell epitopes (Chakhaiyar et al., 2004). as also predicted from hydropathy profile of PPE65, we determined the levels of antibody isotypes in sera of immunized animals. It has been earlier observed that PE protein is less immunogenic when compared to PPE protein, (Tundup et al., 2008). Our findings also suggest the same for PE32, as PPE65 proved to be a good B-cell antigen with a higher antibody response against PPE65 as compared to PE32 in sera of mice immunized with the respective proteins. Analysis of IgG subtypes revealed that PPE65 induces significant increase in IgG1 as compared to IgG2a (IgG1/IgG2a > 1 = Th2 response) levels which predicts a predominance of Th2 type of immune response (Finkelman et al., 1991). This observation was interesting as a number of PE/PPE proteins may be surface exposed and are likely to be targeted by the humoral response and thus dictate the outcome. This was consistent with an earlier observation of another PPE protein Rv2608 which elicited a high humoral and a low T cell response in TB patients (Chakhaiyar et al., 2004). These observations signify the importance of these proteins and the need to explore further to understand the virulence mechanism.

PE/PPE proteins are also known to encompass a number of T cell epitopes and several of them are being assessed as future vaccines. Contrary to this, the dampening effect of PE32/PP65 on Th1 response highlights a different role for this family and also points to its likely involvement in the immune evasion process. Mice immunized with these proteins were found to have reduced production of IFN-γ from splenocytes as a function of protein concentration, though there was increase in the level of IFN-γ with lower concentrations of proteins which could be possibly the result of basal level of interaction of T cells with the antigens. On increasing antigen concentration, the immunomodulatory function comes into play and determines the outcome. To rule out toxicity due to higher concentration of these proteins we performed MTT assay in RAW264.7 cells and found that these proteins are not toxic at 3 μg/ml concentration. IFN-γ is a well-known Th1 cytokine and its production is an important factor involved in protection against TB (Smeltz et al., 2002). This finding also suggests that PE32 and PPE65 suppresses T-cell activity and likely favors Th2 pathway. Th1 response being not the only signature of protection, the importance of Th17 to protection is also emerging. Effect of these PE/PPE proteins on Th1/Th17 immunomodulation will be an interesting aspect of work that remains to be explored.

We also tried to assess for the change in cellular components of splenocytes, as it is known that CD4^+^ T cells are central to the defense and CD8^+^ cells have important role in clearance of pathogens in several diseases including *M. tuberculosis* (van Meijgaarden et al., 2015). We investigated their effect on multifunctional CD4^+^ and CD8^+^ cells expressing IFN-γ and IL-2. There was significant decrease in both antigen specific CD4^+^ and CD8^+^ cells double positive for IFN-γ and IL-2. These poly functional cells are considered as important contributors to protective immunity as these can lyse infected host cells, or can directly kill *M. tuberculosis* by secreting perforin, granzymes, and granulysin (Canaday et al., 2001; Pitabut et al., 2013). The decrease in the number of these poly functional cells was consistent with the earlier findings and ratifies a diminishing Th1 response that in turn escalates pathogen friendly Th2 response. Thus, these PE/PPE proteins seem to be directly involved in dampening of the host immune response in order to help *M. tuberculosis* for its survival.

Our observations suggest that this PE/PPE operon plays distinct and complementary roles in infection process, acting in concert to facilitate adaptation to the hostile host environment. These proteins along with many others could prove to be critical mediators of host responses and determine the outcome of infection.

## Author Contributions

NE and SH conceptualized and designed the research; MK, JS, BS, and SP performed experiments; JS, SP, and MB carried out data analysis; NK contributed in reagents/analytical tools; MK, JS, SH, and NE wrote the manuscript.

## Conflict of Interest Statement

The authors declare that the research was conducted in the absence of any commercial or financial relationships that could be construed as a potential conflict of interest.
