# Supplementary material for: Mycobacterium tuberculosis Co-operonic PE32/PPE65 Proteins Alter Host Immune Responses by Hampering Th1 Response
Source: Front Microbiol. 2016 May 17;7:719. doi: 10.3389/fmicb.2016.00719 (PMC4868851; doi:10.3389/fmicb.2016.00719)
Supplement: Supplementary file 1 [file Image_1.PDF]

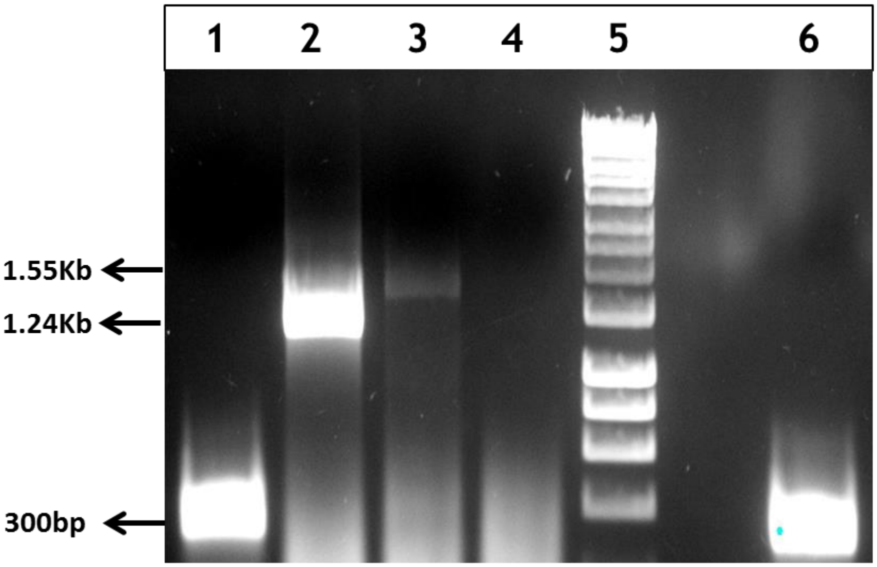

1-*pe32*
2-*ppe65*
3-*pe32+ppe65*
4- -RT (*pe32+ppe65*)
5- DNA ladder
6 – Positive control(*pe32*)

Supplementary figure 1. represents operonic organization of PE32/PPE65 gene pair in RD8 region, reverse primer specific for PPE65 had been used for cDNA synthesis, specific PE32 forward and PE32 reverse primer pairs for amplification PE32(300bp), specific PPE65 forward and specific PPE65 reverse primer pair for the amplification of PPE65 (1.24kb) and forward primer for PE32 and reverse primer for PPE65 for amplification of PE32+PPE65 (1.55kb) had been used. Lane1 shows the amplification of PE32, Lane2 shows the amplification of PPE65 and lane 3 shows the amplification of PE32+PPE65. Lane 4 is negative control (PCR with PE32F and PPE65R, with RNA as template and without Reverse Transcriptase). Lane 6 is positive control (PCR with PE32F and PE32R; genomic DNA of H37Rv was used as template).

Supplementary figure 1
