# Supplementary material for: Mycobacterium tuberculosis Co-operonic PE32/PPE65 Proteins Alter Host Immune Responses by Hampering Th1 Response
Source: Front Microbiol. 2016 May 17;7:719. doi: 10.3389/fmicb.2016.00719 (PMC4868851; doi:10.3389/fmicb.2016.00719)
Supplement: Supplementary file 2 [file Image_2.PDF]

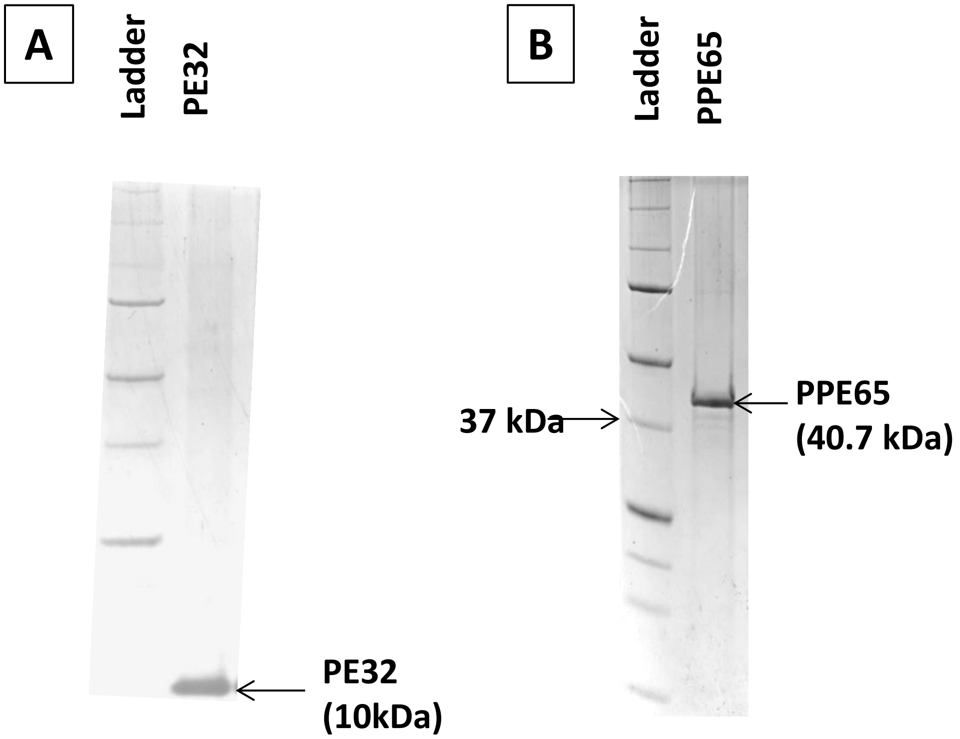

Supplementary Figure 2. PCR amplified genes of respective proteins were cloned in pETDuet-1 vector and transformed into BL21-DE3. Protein expression was induced and purified protein was fractionated on 10% SDS tricine gel depicting purity of **(A)** PE32 and **(B)** PPE65.

Supplementary Figure 2
