# Supplementary material for: Mycobacterium tuberculosis Co-operonic PE32/PPE65 Proteins Alter Host Immune Responses by Hampering Th1 Response
Source: Front Microbiol. 2016 May 17;7:719. doi: 10.3389/fmicb.2016.00719 (PMC4868851; doi:10.3389/fmicb.2016.00719)
Supplement: Supplementary file 3 [file Image_3.PDF]

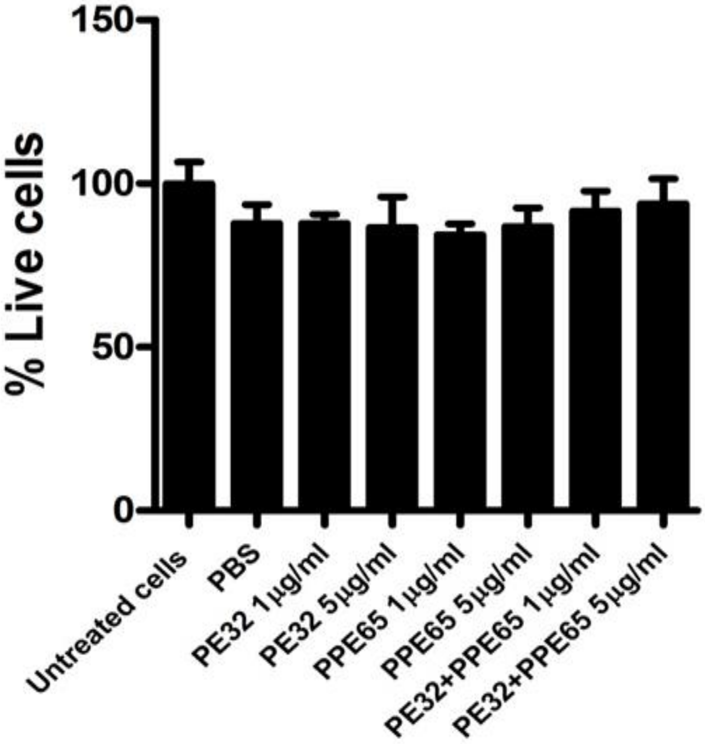

Supplementary Figure 3. MTT assay was carried out in RAW264.7 cells treated with different protein concentration for 24 hours to determine cytotoxicity if any.

Supplementary Figure 3
