# Supplementary material for: Mycobacterium tuberculosis Co-operonic PE32/PPE65 Proteins Alter Host Immune Responses by Hampering Th1 Response
Source: Front Microbiol. 2016 May 17;7:719. doi: 10.3389/fmicb.2016.00719 (PMC4868851; doi:10.3389/fmicb.2016.00719)
Supplement: Supplementary file 4 [file Image_4.PDF]

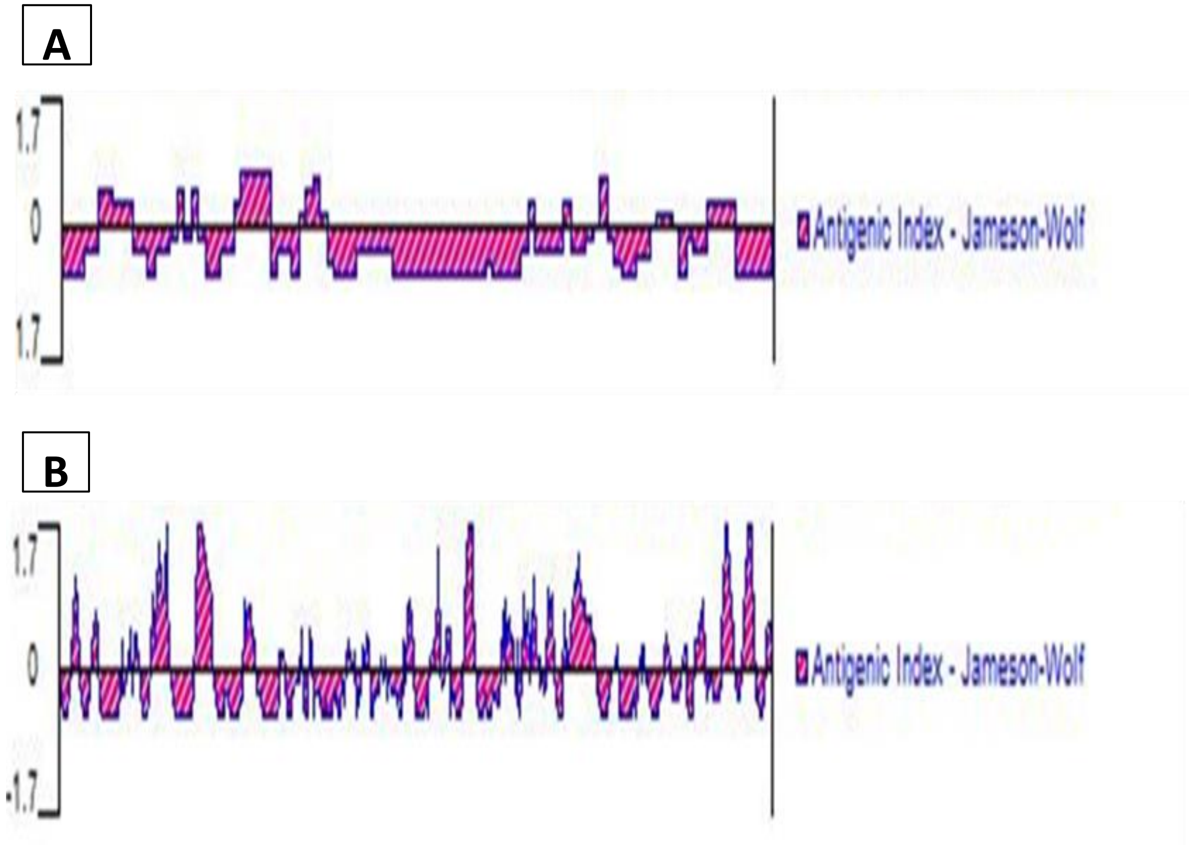

Supplementary Figure 4. Output from DNASTAR depicting antigenic index of **(A)** PE32 and **(B)** PPE65.

Supplementary Figure 4
